# Dasatinib-Loaded Erythrocytes Trigger Apoptosis in Untreated Chronic Myelogenous Leukemic Cells: A Cellular Reservoir Participating in Dasatinib Efficiency

**DOI:** 10.1097/HS9.0000000000000041

**Published:** 2018-06-05

**Authors:** Kelly Airiau, Béatrice Turcq, Stéphane Bouchet, Elodie Laharanne, Jean-Philippe Vial, Gabriel Etienne, François-Xavier Mahon, Francis Belloc

**Affiliations:** 1 INSERM U1218 ACTION, Institut Bergonié, Bordeaux, France; 2 University of Bordeaux, Bordeaux, France; 3 CHU Bordeaux, Bordeaux, France; 4 Institut Bergonié, Bordeaux, France

## Abstract

Dasatinib is an ABL1 tyrosine kinase inhibitor (TKI) with a short in vivo plasmatic half-life but with good efficiency, which is not fully understood. We investigated the possibility that circulating erythrocytes store and then provide dasatinib to target cells. In vitro coincubation of dasatinib-treated cells with naïve leukemic cells followed by analysis of kinase inhibition, apoptosis induction, fluorescent molecule exchanges, and dasatinib dosage were performed. Cells incubated with clinically relevant concentrations of dasatinib for a short time retained, after a washout procedure, an intracellular pool of dasatinib which was transferable to naïve BCR-ABL1 expressing cells and induced their apoptosis. This was verified in total blood where the huge cellular volume of erythrocytes constituted a large reservoir of dasatinib able to induce apoptosis in naïve BCR-ABL1 cell lines and primitive chronic myeloid leukemia (CML) CD34+ cells. This dasatinib transfer necessitated a contact between donor and acceptor cells. A component exchange occurred during this contact, carrying dasatinib and other TKIs such as nilotinib or the fluorescent sunitinib. An active pool of dasatinib could be buried inside the circulating erythrocytes, out of reach of detoxifying mechanisms, but still available for target cells and thus extending the acute effect of the plasmatic pool of the drug.

## Abstract 2

Supplemental Digital Content is available in the text

## Introduction

Chronic myeloid leukemia (CML) is a stem cell hematological disease characterized at the molecular level by the expression of the BCR-ABL1 chimeric oncogenic tyrosine kinase. Tyrosine kinase inhibitors (TKIs) constitute the frontline therapy for CML. Imatinib mesylate, the first TKI to be used for the treatment of CML, has a half-life of about 15 hours^1^ which ensures continuous target inhibition for clinical efficacy. Monitoring TKI inhibition using CRKL phosphorylation as a substrate of BCR-ABL1^2^ and measuring the plasma concentrations of imatinib,^3^ both related to the treatment efficiency, suggest that continuous inhibition is necessary for a clinical response.

To override imatinib resistance, second-generation TKIs were developed with different pharmacological characteristics. Nilotinib exhibits an in vivo half-life similar to imatinib^4^ but, in contrast, dasatinib is characterized by a shorter half-life (3–6 hours) and a higher volume of distribution (3- to 8-fold higher than imatinib or nilotinib).^5,6^ Due to this pharmacological pattern, it was initially administered twice a day in an attempt to maintain target inhibition. However, further clinical investigations demonstrated that once-daily dosing of dasatinib was as efficient and less toxic as twice-daily dosing.^7,8^ Thereafter, in vitro studies showed that a transient inhibition of BCR-ABL1 activity was sufficient to commit CML cells irreversibly to apoptosis^9,10^ even though CRKL phosphorylation was recovered. This effect has been related to the high concentration of TKI (2 log above BCR-ABL1 IC50) which was transiently applied to the cells. While such high doses, above inhibiting concentrations, were not observed in vivo with imatinib treatments, which is active in the micromolar range, they were commonly achieved with second-generation TKIs dasatinib^11^ and nilotinib,^12^ which are supposed to be efficient in the nanomolar range. Subsequently, it had been shown that a pool of TKI was stored in treated cells and that this pool committed cells toward apoptosis in a threshold-dependent manner.^13^ However, because of the short half-life of dasatinib,^5,6^ we questioned whether or not all leukemic cells could be confronted to a sufficient concentration of the drug to reach this threshold level in such a short time.

In the present work, we tested the hypothesis that cells treated at high concentrations of TKIs could transfer a part of their stored TKI to naïve-untreated CML cells. The role of erythrocytes, cells that constitute the largest compartment in the blood, was particularly investigated.

We found that red blood cells stored a significant pool of dasatinib when treated in vitro for a short duration with drug concentrations in the range of the peak ratio found in the plasma of treated patients. This pool was relatively stable after several cell washings. Such a persistent intraerythrocyte reservoir of dasatinib was also found in vivo in the blood of patients under treatment. Moreover, treated erythrocytes transferred a part of this pool to naïve cells triggering apoptosis in dasatinib sensitive cells. A similar transfer of molecules was observed when erythrocytes were labeled with stable fluorescent cell markers or loaded with sunitinib, a fluorescent TKI. Interestingly, a physical contact between donor erythrocytes and acceptor leukemic cells was found to be necessary for the occurrence of this transfer. These results were interpreted and discussed in terms of pharmacokinetic follow-ups of the second-generation TKIs and in terms of their effective availability in the blood of patients.

## Results

### Dasatinib is stored in treated cells

**Figure 1:**
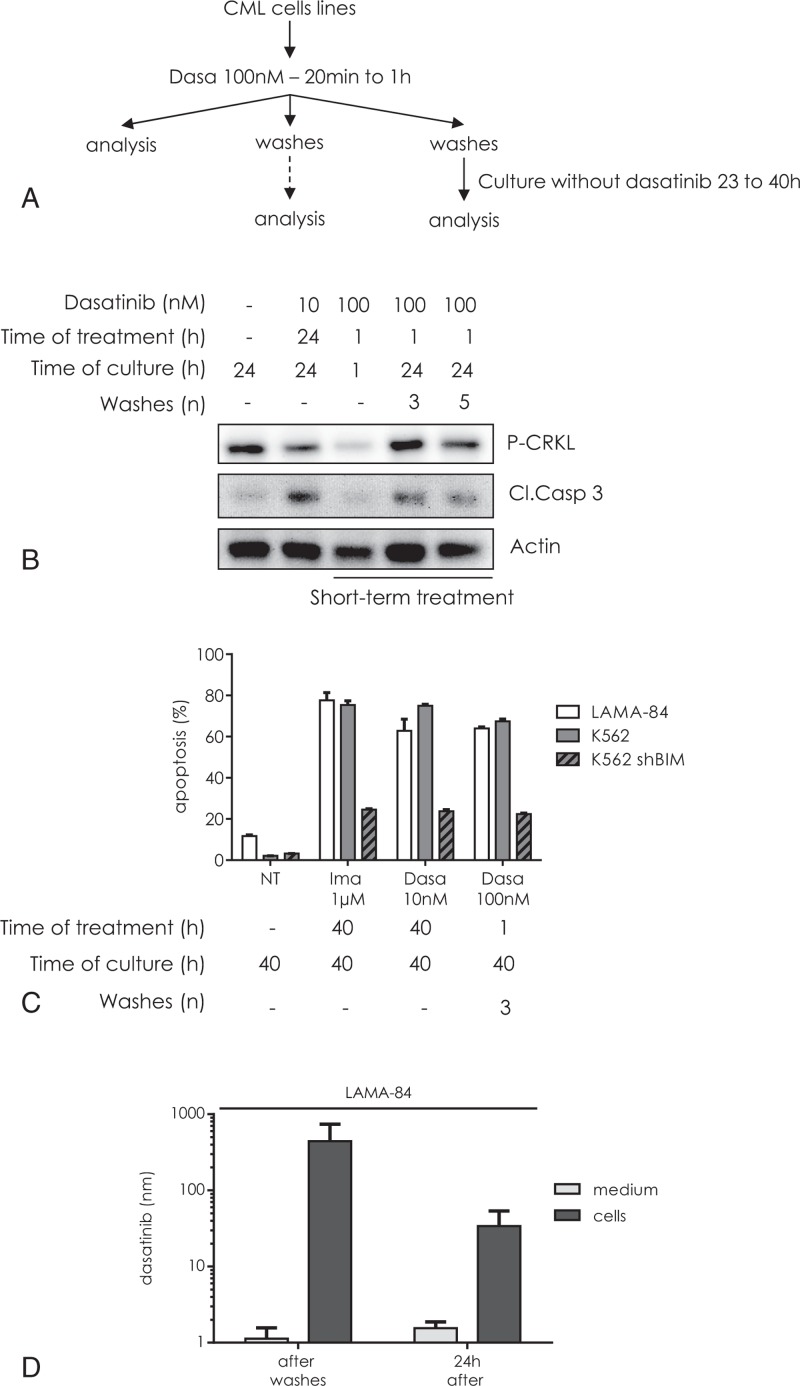
**A short-term treatment with high doses of dasatinib induces a Bim-dependent apoptosis.** (A) Design of the short-term treatment experiment: CML cell lines were treated with dasatinib 100 nM for 20 minutes to 1 hour and immediately analyzed after dasatinib short exposure times. Alternatively, cells were washed for 3 to 5 times and directly analyzed or cultured without dasatinib for indicated time before analysis. (B) LAMA-84 cells were not treated or treated with dasatinib 10 nM for 24 hours. Alternatively, cells were submitted to short-term treatment: after 1 hour exposure to 100 nM dasatinib, cells were directly pelleted or washed 3 or 5 times and further incubated for 23 hours. Total cell lysates were then analyzed by Western blot with antibodies to P-CRKL and cleaved caspase 3 (Cl. Casp 3). Actin was used as a loading control. (C) LAMA-84 (white bars), K562 (gray bars), or K562 depleted in BIM by RNA interference (striped gray bars) were nontreated (NT) or treated with 1 μM imatinib (Ima) or 10 nM dasatinib (Dasa) for 40 hours. CML cells were also submitted to dasatinib short-term treatment (100 nM for 1 hour) and cultured 39 hours in dasatinib-free medium after 3 washes. The percentage of apoptotic cells was measured by flow cytometry using the DiOC_6_(3) probe for MMP. Mean ± SD from 3 independent experiments. (D) LAMA-84 cells were incubated for 20 minutes with 100 nM dasatinib, centrifuged, washed 3 times, resuspended in fresh medium and incubated for 24 hours at 37°C. The dasatinib concentrations were measured in medium (light gray bars) and in cells (dark gray bars) separately, immediately after 20 minutes. incubation, after washout procedure and after 24 hours culturing without dasatinib. The concentration of dasatinib in the cells was calculated assuming a cell volume of 4.2 μL for 10^6^ LAMA-84 cells.

To investigate if a short time incubation of LAMA-84 cells with dasatinib (100 nM for 1 hour—Fig. 1A) could be sufficient to inhibit BCR-ABL1 kinase activity, we analyzed the phosphorylation state of CRKL, a target of BCR-ABL1. A dramatic inhibition of CRKL phosphorylation was observed after short-term dasatinib treatment (Fig. 1B), stronger than those induced by the usual in vitro dasatinib treatment (10 nM—24 hours). When the cells were submitted to short-term treatments, then extensively washed and further incubated for 23 hours without the drug, a partial recovery in CRKL phosphorylation was observed, as compared to cells continuously treated with 10 nM dasatinib. However, these cells underwent as much apoptosis as the continuously treated cells, as evidenced by cleaved caspase 3 analysis and this was not affected by increasing the number of washes.

To confirm this, the percentage of apoptosis was also measured by flow cytometry, after staining with the mitochondrial probe DiOC_6_(3). Cells submitted to the short-term treatment (100 nM dasatinib—1 hour), washed and incubated 39 hours in dasatinib free medium showed similar amounts of cell death than cells submitted to continuous incubation with 1 μM imatinib or 10 nM dasatinib and this for both BCR-ABL1 cell lines assayed (Fig. 1C). Moreover, K562 knock-down for BIM (K562 shBIM) exhibited an apoptotic response decreased by 70% (Fig. 1C) suggesting that this response requires BIM expression. Thus, a short incubation with a high dose of dasatinib was able to further induce apoptosis in a BIM-dependent pathway while BCR-ABL1 activity was partly restored.

It was interesting to verify whether an intracellular pool of dasatinib could be responsible for the delayed apoptotic response previously observed (Fig. 1B and C). LAMA-84 cells were treated with 100 nM dasatinib during 20 minutes, extensively washed to remove the nonincorporated drug and cultured in dasatinib free medium for 24 hours. Dasatinib content was quantified by HPLC in supernatant (medium) and cell pellet (cells), right after 20 minutes incubation with dasatinib, after washing or after 24 hours culturing (Fig. 1D). We found that LAMA-84 cells were able to rapidly concentrate dasatinib from the medium after a short incubation time. This intracellular pool was maintained in spite of the washing steps. Only a small amount of the stored dasatinib was released in the culture medium during the following culture step.

### Erythrocytes store an active pool of dasatinib

**Figure 2:**
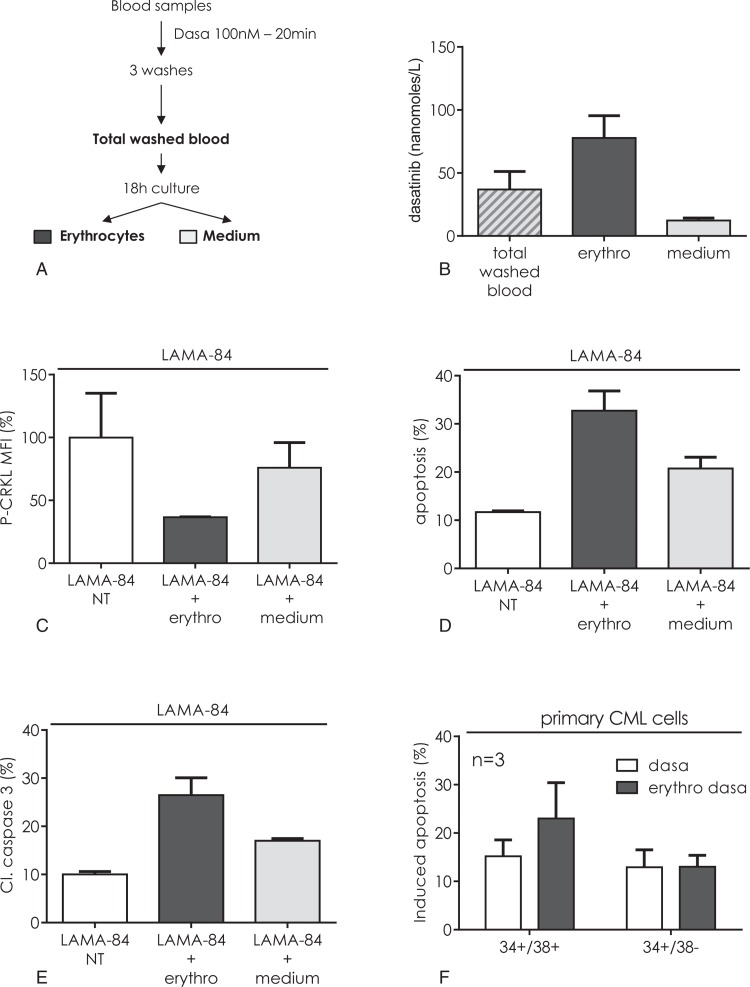
**Blood cells constitute a pool of dasatinib able to induce apoptosis of BCR-ABL1 expressing cells.** (A) Three different blood samples (0.5 mL) were incubated with 100 nM dasatinib for 20 minutes. The samples were centrifuged and washed 3 times with 1 mL of PBS, resuspended in 0.5 mL of culture medium (total washed blood). A part was further cultured for 18 hours and erythrocytes were then separated from the medium by centrifugation. (B) Dasatinib concentration was determined in each fraction. (C–E) CFSE traced LAMA-84 were nontreated (NT) or coincubated with erythrocytes or medium for 24 hours. (C) BCR-ABL1 activity was measured by flow cytometry using anti-P-CRKL antibody. The figure represented the mean fluorescence intensity (MFI) in percentage of nontreated cells (NT). Cell death was measured by flow cytometry using the DiOC_6_(3) MMP probe (D) or anticleaved caspase 3 antibody (E). D shows the percentage of dead cells and E the percentage of cleaved caspase 3 positive cells. Mean ± SD from 3 experiments. (F) Bone marrow mononuclear cells from CML patients were incubated for 24 hours either with 100 nM dasatinib (white bars) or with washed erythrocytes pretreated with 100 nM dasatinib (gray bars). The dasatinib-induced apoptosis was calculated relating to the corresponding untreated cells. The figure shows the dasatinib-induced apoptosis in the CD34+/CD38+ and in the CD34+/CD38− populations. Mean ± SD from 3 different patients.

Owing to the large volume of erythrocytes in the blood, we wondered if short-term, high dose dasatinib treatment of whole blood could be sufficient to create a large storage compartment for the drug. Blood samples were submitted to short-term dasatinib treatment (Fig. 2A). After 20 minutes incubation of blood samples with a clinically relevant concentration (100 nM) of dasatinib and 3 washes to remove the free drug, total washed blood was analyzed or alternatively cultured in a dasatinib-free medium for 18 hours. A part of the drug remained concentrated in the cells and a small part was released into the culture medium (Fig. 2B). This suggested that erythrocytes might serve as a dasatinib reservoir. When erythrocytes were submitted to an osmotic shock, 95% of the stored dasatinib was lost (Suppl. Fig. 5a, Supplemental Digital Content) suggesting that storage occurred in the cytoplasm of erythrocytes. To evaluate the involvement of erythrocytes in the circulating pool of dasatinib, blood was fractionated in plasma, erythrocytes, and platelets after dasatinib treatment. As shown on Suppl. Figure 5b (Supplemental Digital Content), the most amount of dasatinib was found in erythrocytes, even after washing out. Platelets were also able to store a significant amount of dasatinib but this pool was 6-fold lower than the erythrocyte pool.

To verify the inhibitory activity of intraerythrocyte stored dasatinib, dasatinib-loaded erythrocytes were coincubated with naïve BCR-ABL1 expressing LAMA-84 cells. Inhibition of the BCR-ABL1 tyrosine kinase activity was testified by P-CRKL (Fig. 2C). This was associated with cell death induction (Fig. 2D) occurring through an apoptotic pathway (Fig. 2E) demonstrated by caspase 3 cleavage. In parallel, dasatinib released in the medium was also able to inhibit BCR-ABL1 activity, leading to apoptosis. Similarly, dasatinib-loaded erythrocytes induced as much apoptosis in fresh primary progenitor cells from CML patients than direct dasatinib treatment (Fig. 2F).

**Figure 3:**
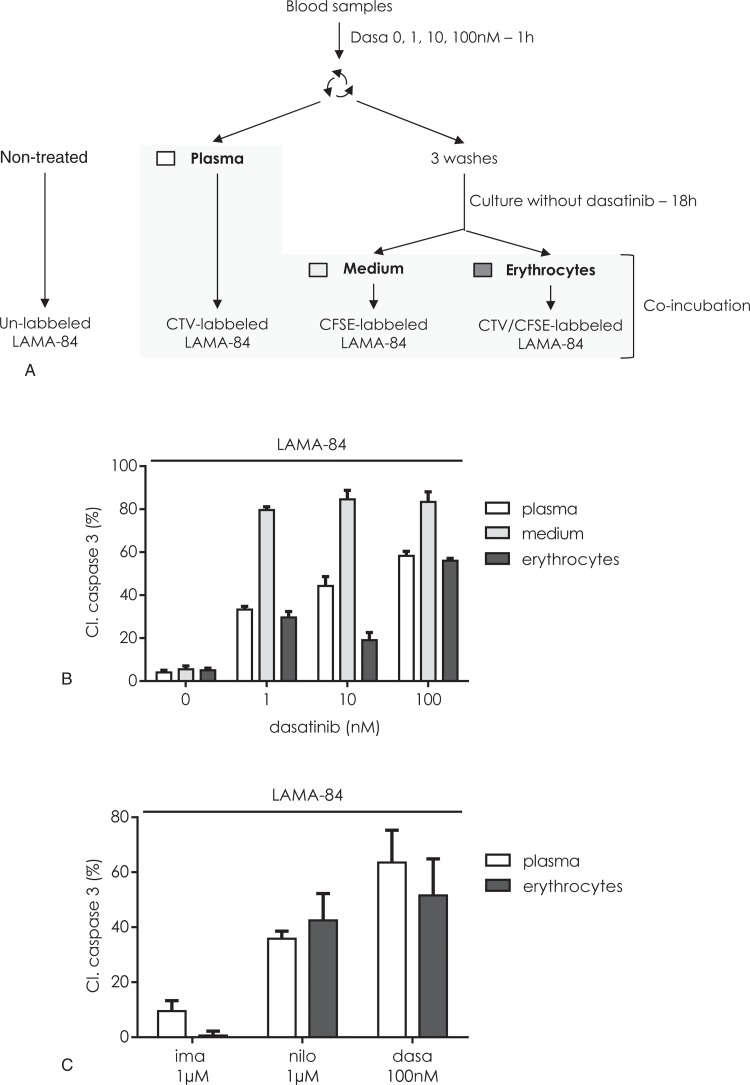
**The constitution of an active pool of TKIs in erythrocytes depends on TKI concentration.** (A) Blood samples were incubated for 1 hour with 0, 1, 10, or 100 nM dasatinib. The plasma was collected by centrifugation; the erythrocyte pellet was washed 3 times and resuspended in culture medium for 18 hours before erythrocytes and medium separation. LAMA-84 cells were either unlabeled or labeled with CFSE, CellTrace Violet (CTV) or both and respectively nontreated or incubated with plasma, medium or erythrocytes. After 24 hours culturing, the four conditions were mixed and analyzed for caspase 3 activation in LAMA-84 cells as described in Materials and Methods Section and in Suppl. Figure 1 (Supplemental Digital Content). (C) The percentage of cleaved caspase 3 positive cells was plotted as a function of nontreated cells. Mean ± SD of 3 different experiments with 3 different blood samples. (C) Blood samples were either nontreated or treated with 1 μM imatinib, 1 μM nilotinib, or 100 nM dasatinib for 1 hours. The plasma was collected by centrifugation and the erythrocytes pellet was submitted to the washout procedure. Plasma (white bars) or washed erythrocytes were then added to CTV-traced LAMA-84 cells and incubation was performed for 24 hours at 37°C. The percentage of cleaved caspase 3 positive cells was analyzed as in B.

The concentration of dasatinib used above was in the maximum concentration range observed in the plasma of patients undergoing dasatinib treatment. To verify whether the pool of intraerythrocyte drug depended on the administrated dasatinib concentration, similar experiments were performed using lower concentrations of this TKI. Blood samples were incubated with different concentrations of dasatinib and LAMA-84 cells were then exposed to treated plasma, erythrocytes or medium (Fig. 3A). An apoptosis-inducing stock of dasatinib was observed with both the medium and the erythrocyte suspension when blood samples were exposed to a concentration of 100 nM dasatinib (Fig. 3B). Exposing erythrocytes to 1 or 10 nM dasatinib failed to induce such amounts of apoptosis. Conversely, for such low concentrations, the corresponding media were able to induce apoptosis. The ability of the medium fraction to induce a higher amount of apoptosis than the plasma fraction implies that dasatinib is quickly stored by erythrocytes, reducing the concentration of available dasatinib in the plasma. The TKI were then released in the medium at a maximum range during the 18 hours culturing. This suggests that the high transitory concentration of dasatinib found in patient plasma, while not necessary to induce immediate apoptosis of target cells, could generate an extra pool of drug stored in part by erythrocytes, thus constituting an intracellular, long life reservoir of the drug. In a long-term experiment, we confirmed that dasatinib-loaded erythrocytes were able to eradicate almost all the LAMA-84 cells in vitro (Suppl. Fig. 2, Supplemental Digital Content).

It was interesting to verify if such a pool could be obtained with other CML related TKI. We assayed the apoptosis-inducing ability of plasma and erythrocytes from blood treated with clinically relevant concentrations of imatinib and nilotinib compared to dasatinib (Fig. 3C). It was found that both second-generation TKI (i.e., nilotinib and dasatinib) generated an active intraerythrocyte drug reservoir, while imatinib did not. It must be noticed that imatinib blood concentration is in the range of the IC50 concentration while the blood concentrations of nilotinib and dasatinib were 2 logs higher than their respective IC50.

### Physical contact between erythrocytes and target cells is necessary to bolster the toxicity of the erythrocyte dasatinib pool

**Figure 4:**
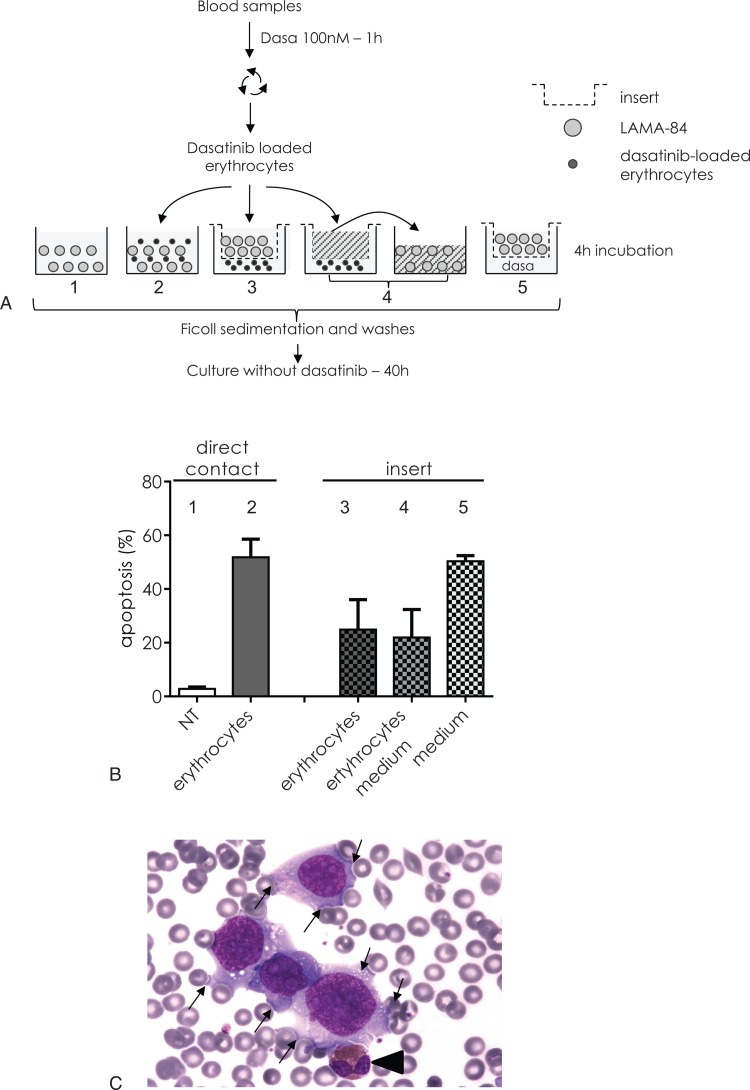
**Dasatinib-loaded erythrocytes induce apoptosis of leukemic cells through a contact-dependent mechanism.** (A) Design of the experiment: Blood samples were submitted to dasatinib short-term treatment as previously described. CTV-traced LAMA-84 cells were nontreated (1) or incubated in contact with dasatinib-loaded erythrocytes (2). Alternatively, CTV-traced LAMA-84 cells were incubated in a culture insert with dasatinib-treated erythrocytes in the lower compartment (3) or with medium which has been previously incubated for 4 hours with treated erythrocytes through a culture insert (4). In a fifth well, CTV-traced LAMA-84 cells were incubated in a culture insert with 100 nM dasatinib-containing medium in the lower compartment. The LAMA-84 cells were centrifuged on a Ficoll cushion to eliminate contaminating erythrocytes, washed and further incubated at 37°C. (B) After 40 hours of culture, LAMA-84 apoptosis was measured by flow cytometry using DiOC_6_(3) as MMP probe. Mean percentage of apoptotic cells ± SD of 3 independent experiments with 3 different bloods. (C) LAMA-84 cells were added to normal blood and a smear was stained using May-Grunwald-Giemsa. Contacts between erythrocytes and LAMA-84 cells can be observed (arrows). A normal eosinophil leucocyte is shown (arrowhead).

To assess the nature of dasatinib exchanges between dasatinib-loaded erythrocytes and naïve CML cells, the effect of coincubation in direct contact or through a culture insert was investigated (Fig. 4A). When dasatinib was directly added in the medium and separated from the LAMA-84 cells by a membrane of 3 μm porosity, the apoptotic rate was similar to dasatinib in solution (Fig. 4B—fifth bar/Fig. 1C). When incubated 4 hours in the presence of LAMA-84 cells, dasatinib-loaded erythrocytes were able to further induce apoptosis in leukemic cells (Fig. 4B—second bar) as efficiently as dasatinib in solution (Fig. 1C). This cell death rate was reduced by 55% when both cell populations were separated by a culture insert (Fig. 4B—third bar). When dasatinib-loaded erythrocytes were replaced by their incubation medium on the other side of the insert, the rate of apoptosis in LAMA-84 cells was as much as LAMA-84 incubated with dasatinib-loaded erythrocytes through the insert membrane (Fig. 4B—fourth bar). This last result suggests that the diffusion of dasatinib outside the erythrocytes membrane accounts for 45% of apoptotic events. However, 55% of the apoptosis-inducing effect of dasatinib-loaded erythrocytes did not occur when the physical contact between erythrocytes and responsive LAMA-84 was impeded. Such a contact between erythrocytes and leukemic cells was frequently observed when leukemic cell lines were added to blood before microscopic examination (Fig. 4C). It was interesting to verify if such a contact between donor and acceptor cells was accompanied by material transfer from one cell to another one.

### Erythrocytes are able to transfer components to acceptor leukemic cells

**Figure 5:**
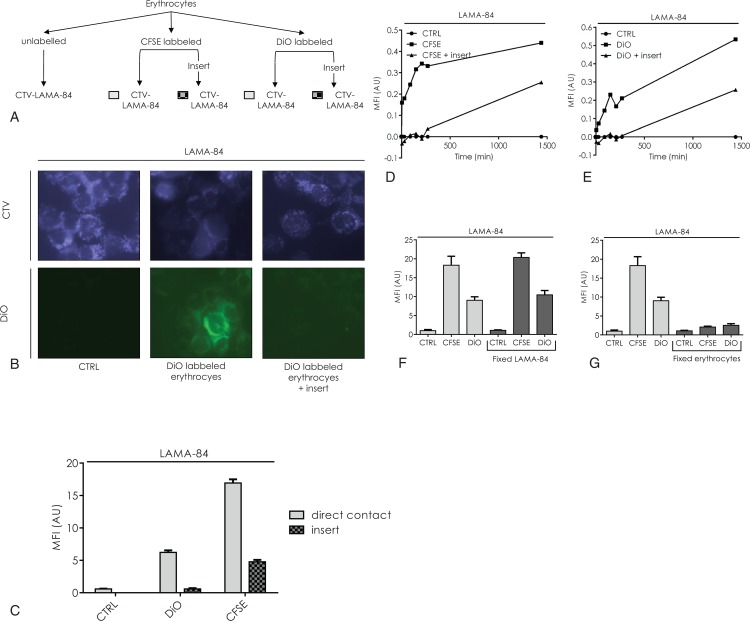
**Erythrocytes are able to transfer components to leukemic cells during contact.** (A) Design of the experiment B and C: CTV-traced LAMA-84 cells were incubated either in the presence of unlabeled erythrocytes (CTRL) or in the presence of erythrocytes whose membranes were labeled with DiO in direct contact (DiO) or through a culture insert (DiO—inset). Alternatively, CTV-traced LAMA-84 were incubated with erythrocytes whose plasma was labeled with CFSE in direct contact or through a culture insert. (B) After 1 hour incubation, the erythrocytes were eliminated by Ficoll centrifugation and CTV-traced LAMA-84 sedimented on a slide by cytospin. The fluorescences were microscopically observed through a blue filter for CTV (upper panel of photographs) and a green filter for DiO (lower panel of photographs). (C) Unlabeled or labeled erythrocytes were incubated with CTV-traced LAMA-84 cells for 1 hour at 37°C, in direct contact (gray bars) or through a culture insert (checkered bars) and analyzed by flow cytometry for green fluorescence after gating on the blue fluorescent LAMA-84 cells. The mean green fluorescence intensity (MFI) was plotted as a function of the label. Mean ± SD of 3 samples from different bloods (white bars). (D and E) Unlabeled (circle—control sample) or labeled erythrocytes were incubated in the presence of CTV-traced LAMA-84 cells for different times in direct contact (squares) or through a culture insert (triangles). Green fluorescence of LAMA-84 cells was analyzed by flow cytometry for each incubation time. The MFI of the control sample was subtracted from the MFI of the test sample for each time point. (F and G) LAMA-84 cells were incubated with unlabeled (CTRL), DiO or CFSE labeled erythrocytes for 1 hours at 37°C. The transfer of fluorescence was measured by flow cytometry as above. When indicated fixed LAMA-84 cells (F) or fixed erythrocytes (G) were compared to unfixed cells. Mean ± SD of 3 different experiments with different blood samples.

This hypothesis was tested by labeling the erythrocytes with stable tracers: the lipophilic DiO incorporated in plasma membranes and the CFSE incorporated in cytoplasm (Fig. 5A). When labeled erythrocytes were incubated in the presence of LAMA-84, a part of the membrane (DiO labeled) was transferred to the leukemic cells but this only occurred when a physical contact was possible (Fig. 5B and C). Similar cytoplasm transfers were much stronger when a direct contact was possible (Fig. 5C). A kinetic experiment showed that for both labels, the transfer of fluorescence occurred in a 2 mode curve. Firstly, a rapid saturable transfer was observed during the first 2 hours followed by a further linear incorporation of fluorescence which was prolonged over 24 hours (Fig. 5D and E). When donor and acceptor cell populations were separated by a porous membrane, only the second phase of incorporation was observed, suggesting that it was due to passive diffusion of the labels out of the erythrocytes. Fixation experiments (Fig. 5F and G) showed that erythrocytes were active partners in the exchange of components whereas LAMA-84 cells passively incorporated the labeled tracers. Similarly, erythrocytes were able to transfer lipophilic molecules such as DiO to adhering cells such as HeLa cells in a time- and concentration-dependent manner (Suppl. Fig. 3, Supplemental Digital Content). However, these stable fluorescent labels are molecularly very different to TKI.

### Erythrocytes are able to transfer TKI to acceptor leukemic cells in a contact-dependent manner

**Figure 6:**
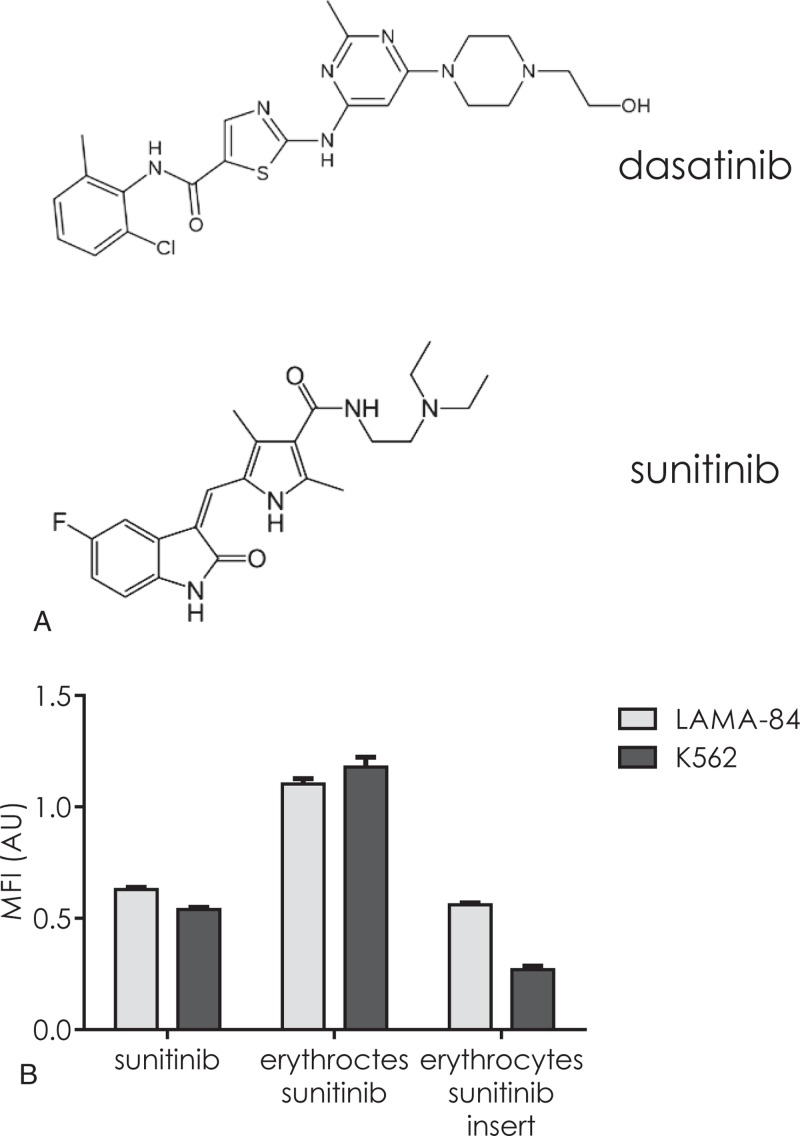
**Erythrocytes are able to transfer TKI to target leukemic cells.** (A) Compared molecular structures of dasatinib and sunitinib. (B) Erythrocytes were loaded with sunitinib by incubation for 1 hour with 1 μM sunitinib and extensive washing. CTV-traced K562 (dark gray bars) or CTV-traced LAMA-84 (light gray bars) cells were incubated with 1 μM sunitinib for 1 hour and submitted to washout procedure (sunitinib). Similarly, CTV-traced K562 or LAMA-84 were incubated with sunitinib-loaded erythrocytes for one hour (erythrocytes sunitinib) in a same well or separated by a culture insert (erythrocytes sunitinib insert). The green fluorescence of leukemic cells was measured by flow cytometry. The MFI of control untreated cells were subtracted from the MFI of each measurement. Mean ± SD of 3 different experiments with different blood samples.

To assess whether erythrocytes induce apoptosis of target cells by transferring TKI to naïve cells, we took advantage of the fluorescence properties of sunitinib. This TKI is not an inhibitor of BCR-ABL1 but shares structural similarities with dasatinib (Fig. 6A) and can easily be analyzed by flow cytometry due to its green fluorescence. When sunitinib-loaded erythrocytes were incubated with naïve K562 or LAMA-84 cells, a transfer of the molecule was objectified by fluorescence measurement inside the leukemic cells (Fig. 6B). Here again, the physical separation of donor and acceptor cell populations by porous membrane resulted in a 49% and 77% decrease in TKI transfer for LAMA-84 and K562 cells, respectively. The amount of transferred TKI was dependent on both the sunitinib burden in each erythrocytes and the erythrocyte density in the coincubation medium (Suppl. Fig. 4, Supplemental Digital Content).

### An intraerythrocyte dasatinib pool does exist in vivo

**Figure 7:**
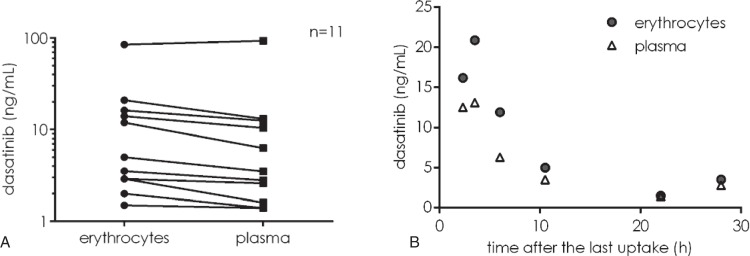
**An intraerythrocyte pool of dasatinib is circulating in vivo.** (A) Dasatinib concentration was measured separately in the plasma and cell fractions of the blood from 11 CML patients under dasatinib treatment. The figure shows the dasatinib concentration in ng/mL in the erythrocytes and in the plasma. (B) For 7 of these patients treated with 40, 50, or 60 mg/day of dasatinib, the duration since the last uptake of the drug was known. The dasatinib concentration (ng/mL) was plotted as a function of the time (hours) after the last uptake for the erythrocytes (gray circle) and the plasma (white triangle) fractions.

The above-described experiments were performed after in vitro loading of erythrocytes with dasatinib. It was necessary to verify if such an intraerythrocyte reservoir of dasatinib circulated in the blood of patients during dasatinib treatment. Dasatinib concentration was measured separately in the plasma and erythrocyte fractions from the blood of patients during their treatment with dasatinib. As shown in Fig. 7A, significant amounts of the drug were found in all the erythrocyte fractions. Moreover, the concentration of dasatinib was higher in the erythrocytes than in the plasma fraction in all but one sample. A kinetic analysis showed that this was true at any time of the day after dasatinib intake (Fig. 7B) and that the kinetics of drug half-life was similar in the 2 fractions probably due to, the continuous, passive diffusion of the drug outside the erythrocytes.

## Discussion

In this work, we showed that a short-term exposure to high concentrations of dasatinib was sufficient to further induce apoptosis of CML cells. This was observed 24 hours later in spite of a partial recovery of BCR-ABL1 signaling. These results have been previously interpreted in terms of oncogenic shock^9,10^: the drug would not be useful anymore afterwards. Conversely, it has been suggested that a residual threshold concentration of dasatinib could be responsible for this induction of apoptosis^13^ and the concentrations of dasatinib in the medium and the cellular compartments described here are in the same range as the previously measured concentrations.^13^ However, we demonstrated that cells which are transiently exposed to high concentrations of dasatinib were also able to induce apoptosis in other unexposed cells. The proapoptotic signal is supplied by dasatinib itself, which was stored in these exposed cells during a short-term treatment and then distributed to naïve cells. It is interesting that although dasatinib remains inside the cell at a concentration much higher than the IC50 of the kinase (Fig. 1D), a recovery in BCR-ABL1 activity, as assessed by CRKL phosphorylation, was observed (Fig. 1B).^2,9,10^ It seems that the cells are able to adapt to the presence of the TKI in terms of enzyme activity but died anyway. Moreover, as described for other TKIs,^14–16^ we demonstrate that dasatinib-induced apoptosis necessitates BIM expression.

The ability of treated cells to store dasatinib is of particular importance. Erythrocytes, the most abundant cell type in the blood (45 to 55% hematocrit), can efficiently accumulate these TKI and effectively act as reservoirs for the drug. In our experiments, a 86% partition coefficient was found for dasatinib (Fig. 2B) while it is around 50% for imatinib.^17^ It must be noticed that unlike imatinib, dasatinib cellular uptake occurs freely, independently of OCT-1 activity.^18,19^ It must be noticed that besides erythrocytes and leukemic cells, other blood cells are able to store dasatinib. We found that blood platelets, for example were able to store a significant amount of dasatinib. This amount represents 6-fold less dasatinib than the amount in circulating erythrocytes (Suppl. Fig. 5b, Supplemental Digital Content) while the circulating platelet compartment volume was about 200-fold lower than the erythrocyte compartment. The ability of erythrocytes to act as a dasatinib pool must also be related to their huge number. This implies that variations in the hematocrit in vivo could result in variations in the dasatinib bioavailability. We also found (Suppl. Fig. 5a, Supplemental Digital Content) that dasatinib was stored in the cytoplasmic fraction of erythrocytes but the mechanism of storage was not elucidated yet.

Moreover, our results indicate that, while the dasatinib-exposed cells release a part of the stored drug in the culture medium, they are probably capable of delivering higher amounts after contact with unexposed cells: as shown in Figure 4B. This suggests that blood cells could provide a dasatinib rich environment to target cells while maintaining a large reservoir of the drug out of reach of the detoxification mechanisms in vivo. Interestingly, though not completely understood, the mechanism of dasatinib transfer from erythrocytes to leukemic cells depends on a contact between donor and acceptor cells and the transfer of components, including the drug itself, occurs during this cell–cell contact. This mechanism can be linked to trogocytosis which has been previously described between red blood cells and monocytes leading to a synaptic transfer of components.^20^ However, the process described here is independent of antibody recognition but is due to the high probability that erythrocytes, due to their huge number, will encounter target cells in the blood. Here, we showed that an increase in the ratio of erythrocytes/LAMA-84 cells correlated with an increase of transferred sunitinib from erythrocytes to target LAMA-84 cells. Equally, we showed that sunitinib transfer was already efficient at ratios ranging from 10 to 160, even if these ratios are several hundred folds lower than those in the blood (Suppl. Fig. 4, Supplemental Digital Content).

This drug delivery mechanism is not specific for dasatinib and was also found for the second-generation TKI nilotinib, but is deemed clinically more significant for dasatinib regarding its short lifetime in plasma. However it was not found efficient for the first-generation TKI imatinib at clinically relevant concentrations. This discrepancy between first- and second-generation TKI could be explained by the efficient doses of the different drugs. The 2 second-generation molecules were efficient to inhibit BCR-ABL1 at low concentrations (in the nanomolar range) while imatinib requires far higher concentrations (micromolar range). However, while the peak concentrations encountered in the plasma of treated patients were equivalent to efficient concentration for imatinib, they were about 100 times higher than the useful dose for both second-generation TKIs. This could favor the accumulation of a persistent intracellular pool of TKI in the case of dasatinib and nilotinib, which is then progressively provided to other target cells.

Moreover, the dasatinib treated cells are able to store the drug for quite a long-time in vitro (days, see Figs. 1D and 2B) as compared to the related short half-life of the drug in vivo (hours). This means that when in the blood, the TKI can be brought almost anywhere in the organism by erythrocytes and delivered to any cell able to accept it for a longer duration than previously supposed. Although erythrocytes are not supposed to reach in close contact with leukemic precursors in the bone marrow, they could maintain a dasatinib-rich environment during passage through capillary vessels. Such a transfer from blood toward bone marrow however would necessitate the participation of the endothelial cells of the vessel wall. Actually, the measurements we performed in the blood of treated patients showed that, in vivo, the clearance of dasatinib was equivalent in the red blood cell and plasma fractions. It seems that at least half of the drug burden remains stored in the erythrocytes. Usually the clearance of the drug was measured in the plasma of the treated patients, but in the light of our results, it could be more appropriate to measure clearance in the whole blood, including the cell fraction. It has been previously suggested^13^ that consideration and monitoring of drug retention inside target cells will yield complementary data for interpreting and defining a rationale behind optimal dosing regimens for CML treatments. In view of our results, the estimation of the intraerythrocyte pool of TKI would be also of critical importance and relatively easy to consider in this field. It has been reported that dasatinib was a substrate for the ABCG2 transporter which was responsible for its efflux from the cells.^18,21^ ABCG2 expression on the erythrocyte membrane can vary from one patient to another^22^ and thus could influence the concentration of dasatinib in the cellular pool. Finally, such a large cellular pool of deliverable drug could be accounted for in clinical results showing that a treatment with only one 100 mg daily dose of dasatinib is as efficient as two 70 mg doses in spite of the supposed very short half life of the drug.^7,8^ Additionally, drug delivery by erythrocytes has been previously described for several drugs^23,24^ but here it is described for the first time concerning TKIs. We also show that this mode of delivery can concern several TKIs (including nilotinib and sunitinib) and not only in the field of hematology. Consequently, the ability to be stored and delivered by erythrocytes could be a new screening parameter to take into account when designing and developing next-generation TKIs.

## Materials and methods

### Reagents

Nilotinib (Tasigna, Novartis, Switzerland) and imatinib (Glivec, Novartis, Bâle, Switzerland) were purchased from Selleck. Dasatinib (Sprycel, Bristol-Myers Squibb, Rueil-Malmaison, France) was kindly provided by BMS.

### Cells and patients

K562 and LAMA-84 cell lines were cultured in RPMI 1640 supplemented with 10% (v/v) fetal calf serum (FCS), 1 mM glutamine, 25 mM HEPES, 100 units/mL penicillin, 50 μg/mL streptomycin in a humidified atmosphere containing 5% (v/v) CO_2_ at 37°C. Exponentially growing cells were used in all experiments. K562 depleted in BIM (K562 shBIM) were obtained by RNA interference as previously described.^16^

After informed consent was obtained, bone marrow aspirates from CML patients which were received in the laboratory for BCR-ABL1 analysis were separated by Ficoll sedimentation. The mononuclear cell layer was washed, and resuspended in culture medium.

For coincubation experiments, total blood samples were from informed healthy donors and used as indicated in coincubation experiments.

For in vivo dasatinib measurement, blood was collected from 11 dasatinib-treated CML patients on heparin-anticoagulated vacutainer tubes by venipuncture and erythrocytes were decanted from plasma by centrifugation.

### Short treatment and washout procedure

Except when notified, the cells were incubated in culture medium at 37°C for 20 minutes to 1 hour with 100 nM dasatinib or other molecules under investigation at the indicated concentrations. Cells were then washed 3 times with 10 volumes of culture medium by centrifugation and resuspended in culture medium without the drug.

### Western blot

After SDS–PAGE electrophoresis, proteins were transferred onto a PVDF membrane (Biorad, Marnes-la-Coquette, France). Membranes were saturated with 5% (w/v) fat-free dry milk or 5% (w/v) bovine serum albumin in Tris-buffered saline containing 0.1% (v/v) Tween 20 (Sigma, Saint-Louis, Missouri, USA). Membranes were then probed with primary antibodies: rabbit monoclonal for cleaved caspase 3, or phospho-CRKL (Y207) (Cell Signaling Technology, Inc., Danvers, Massachusetts, USA), and rabbit polyclonal antibody for actin (Sigma). All of them were used at a 1/1000 dilution. After secondary antibody labeling (Jackson Immuno Research Laboratories, West Grove, Pennsylvania, USA), peroxidase activity was revealed using the Western Lightning Plus-ECL kit (Perkin Elmer, Courtaboeuf, France) and band intensity was quantified using a Kodak Imager.

### Cell death measurements

BCR-ABL1 cell lines death was analyzed by flow cytometry detection using DiOC_6_(3) (100 ng/mL) as a probe to monitor mitochondrial membrane potential (MMP) as previously described.^25,26^ Samples were analyzed with a Navios cytometer (Beckman–Coulter, Villepinte, France) with a blue excitation and a green detection.

Bone marrow mononuclear cells were stained with APC-anti CD34, PC7-anti CD38 and FITC-annexin V for apoptosis labeling before flow cytometry analysis. The percentage of annexin V positive cells of CD34+-CD38+ and CD34+-CD38− populations were extracted for each condition as previously described.^27,28^ Dasatinib-induced apoptosis was calculated using either nontreated cells or cells incubated with nontreated erythrocytes as controls.

In some experiments, when CML cell lines or primary cells were not traced with CTV, Ficoll sedimentation was performed to remove erythrocytes and isolate CML cells thus facilitating the flow cytometry analysis.

### Flow cytometry analysis of inhibitory effects of TKI

The cells were fixed for 10 minutes with 4% (v/v) formaldehyde, permeabilized with 0.1% (v/v) Triton X-100 and postfixed with 50% (v/v) methanol. The cells were then stored at −20°C until use. Before labeling, 5 × 10^5^ cells were centrifuged, washed in PBS containing 3% (w/v) bovine serum albumin (PBS-BSA) and incubated with either anti-phospho CRKL or anticleaved caspase 3 antibodies (Cell Signaling Technology, Inc.) at a 1/100 dilution overnight at 4°C. After washing in PBS-BSA, cells were labeled with an antirabbit IgG antibody coupled to Alexa 640 (Life Technologies, Invitrogen, Saint Aubin, France) at a 1/1000 dilution for 2 hours at 20°C. Cells were washed and resuspended in PBS-BSA before flow cytometry analysis (Suppl. Fig. 1, Supplemental Digital Content). The permeabilization step induced erythrocyte lysis and the Ficoll sedimentation step was thus unnecessary.

### Dasatinib measurement

Supernatant medium analyses were performed using solid-phase extraction (SPE) and tandem mass spectrometry coupled with chromatography (LC-MS/MS), with chromatographic and detection conditions as previously described.^3^ This method has been adapted for the analysis of blood by a previous precipitation with acetonitrile and for cells by a dilution. The calibration ranged from 0.1 to 200 ng/mL (0.205–410 nM). The volume of K562 cells was calculated, assuming the cells as 20 μm diameter spheres.

For in vivo dasatinib measurement, blood was collected from 11 dasatinib-treated CML patients on heparin-anticoagulated vacutainer tubes by venipuncture and erythrocytes were decanted from plasma by centrifugation. Four patients were treated with dasatinib 100 mg/day, 1 with 40 mg/day, 3 with 50 mg/day and 3 with 60 mg/day.

## Supplementary Material

Supplemental Digital Content
