# Supplementary material for: Dasatinib-Loaded Erythrocytes Trigger Apoptosis in Untreated Chronic Myelogenous Leukemic Cells: A Cellular Reservoir Participating in Dasatinib Efficiency
Source: Hemasphere. 2018 Jun 5;2(3):e41. doi: 10.1097/HS9.0000000000000041 (PMC6745996; doi:10.1097/HS9.0000000000000041)
Supplement: Supplemental Digital Content [file hs9-2-e41-s001.ppt]

## Slide 1
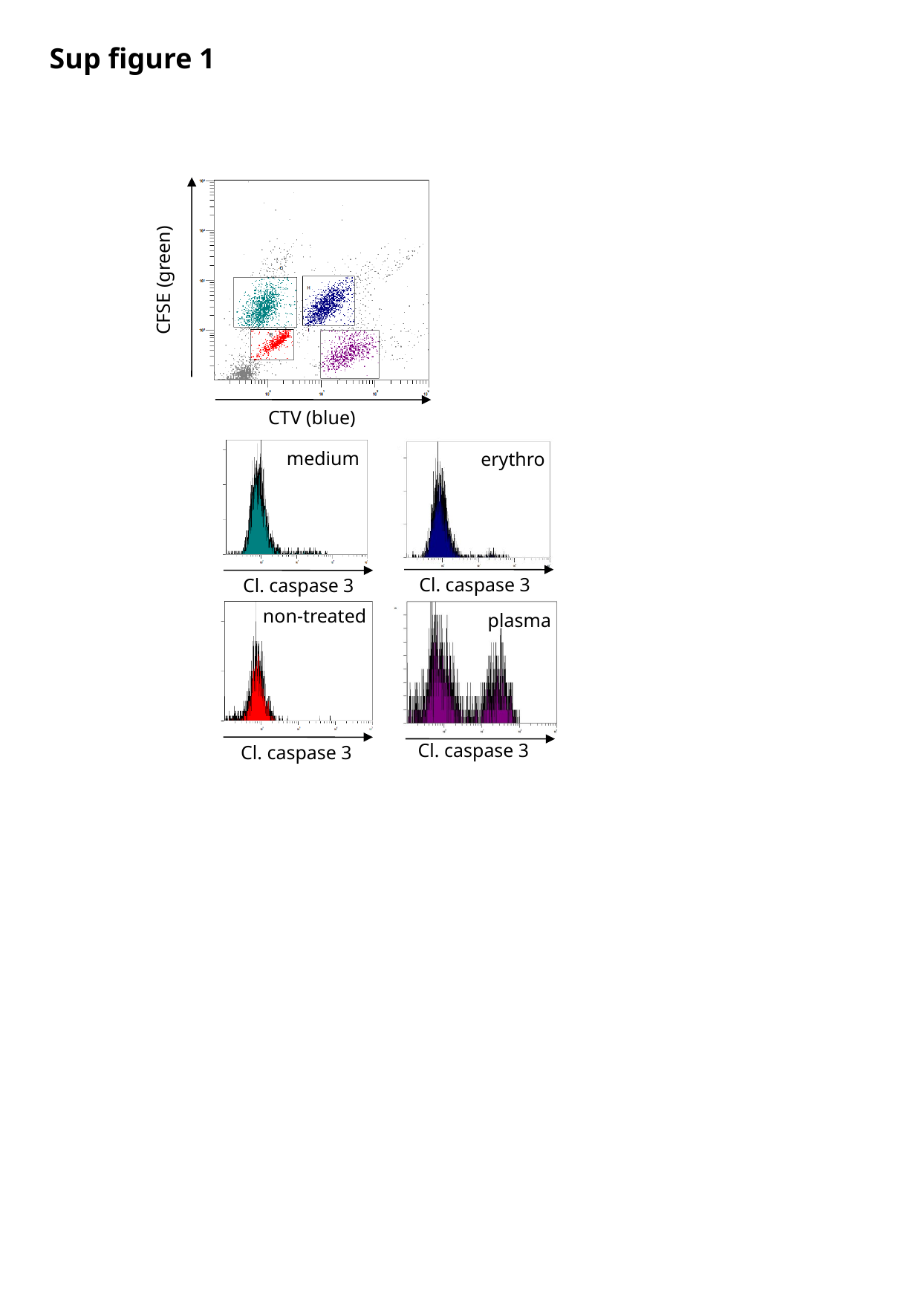

Sup figure 1
CFSE (green)
CTV (blue)
medium
erythro
Cl. caspase 3
Cl. caspase 3
non-treated
plasma
Cl. caspase 3
Cl. caspase 3

## Slide 2
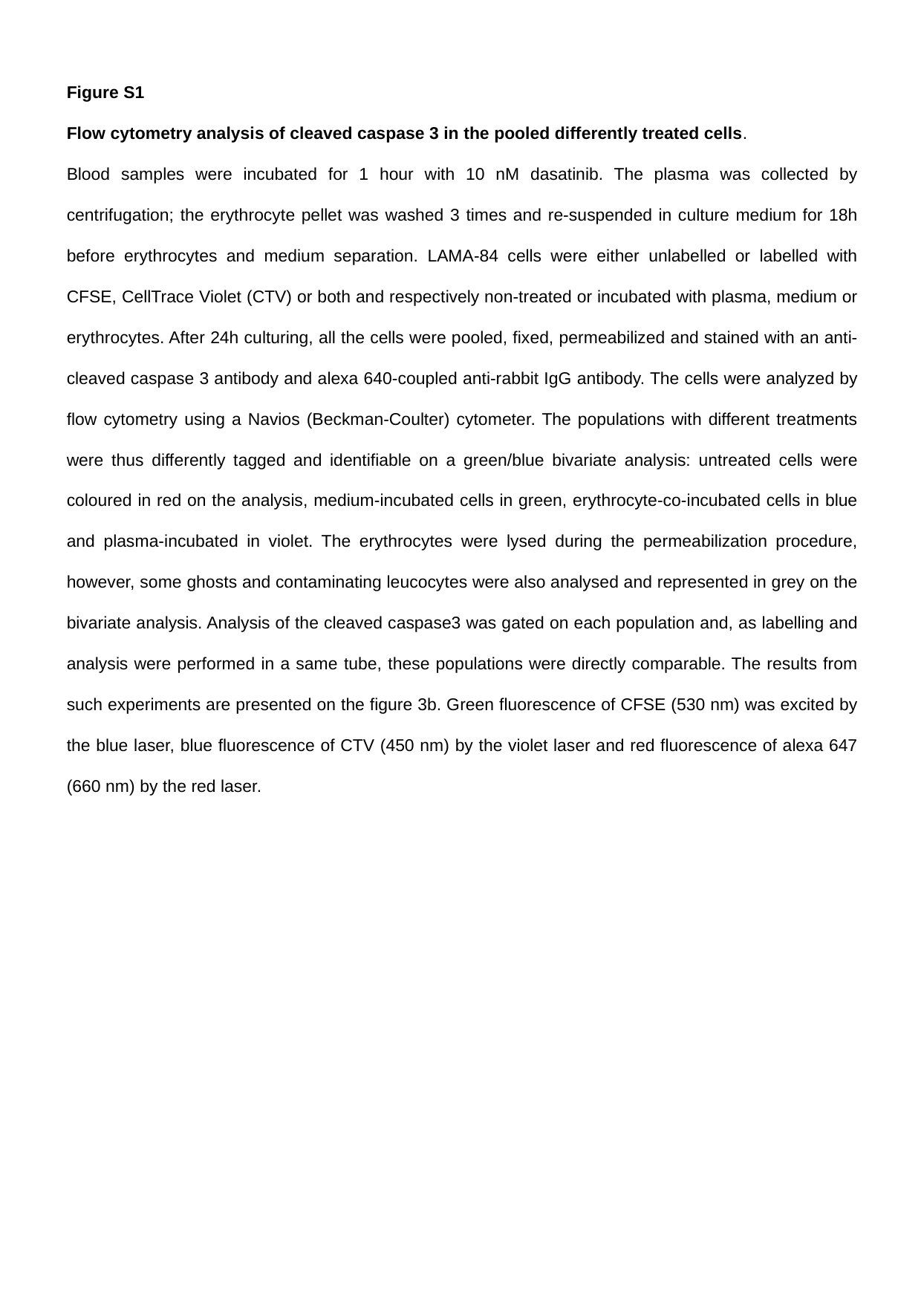

Figure S1
Flow cytometry analysis of cleaved caspase 3 in the pooled differently treated cells.
Blood samples were incubated for 1 hour with 10 nM dasatinib. The plasma was collected by centrifugation; the erythrocyte pellet was washed 3 times and re-suspended in culture medium for 18h before erythrocytes and medium separation. LAMA-84 cells were either unlabelled or labelled with CFSE, CellTrace Violet (CTV) or both and respectively non-treated or incubated with plasma, medium or erythrocytes. After 24h culturing, all the cells were pooled, fixed, permeabilized and stained with an anti-cleaved caspase 3 antibody and alexa 640-coupled anti-rabbit IgG antibody. The cells were analyzed by flow cytometry using a Navios (Beckman-Coulter) cytometer. The populations with different treatments were thus differently tagged and identifiable on a green/blue bivariate analysis: untreated cells were coloured in red on the analysis, medium-incubated cells in green, erythrocyte-co-incubated cells in blue and plasma-incubated in violet. The erythrocytes were lysed during the permeabilization procedure, however, some ghosts and contaminating leucocytes were also analysed and represented in grey on the bivariate analysis. Analysis of the cleaved caspase3 was gated on each population and, as labelling and analysis were performed in a same tube, these populations were directly comparable. The results from such experiments are presented on the figure 3b. Green fluorescence of CFSE (530 nm) was excited by the blue laser, blue fluorescence of CTV (450 nm) by the violet laser and red fluorescence of alexa 647 (660 nm) by the red laser.

## Slide 3
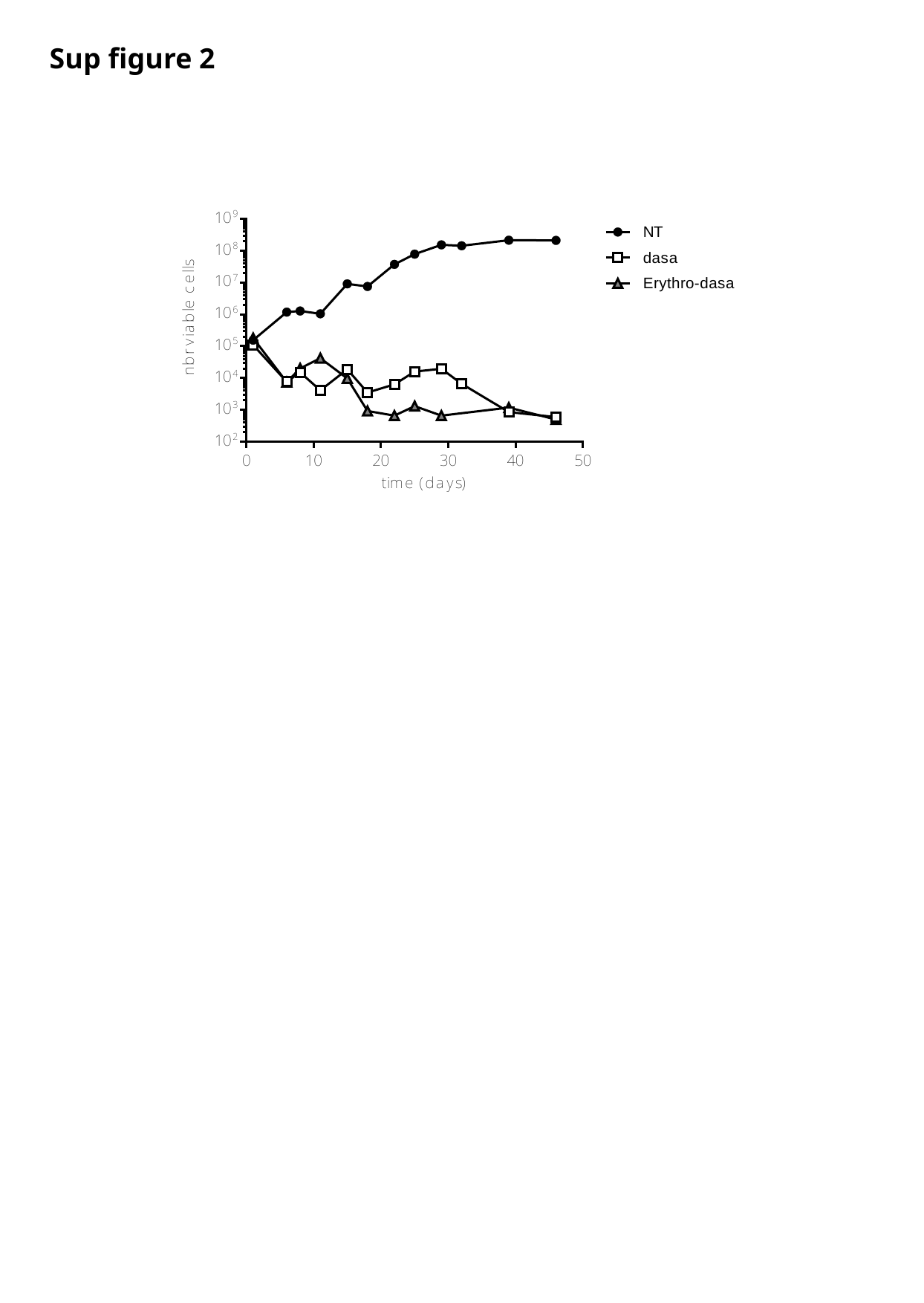

Sup figure 2

## Slide 4
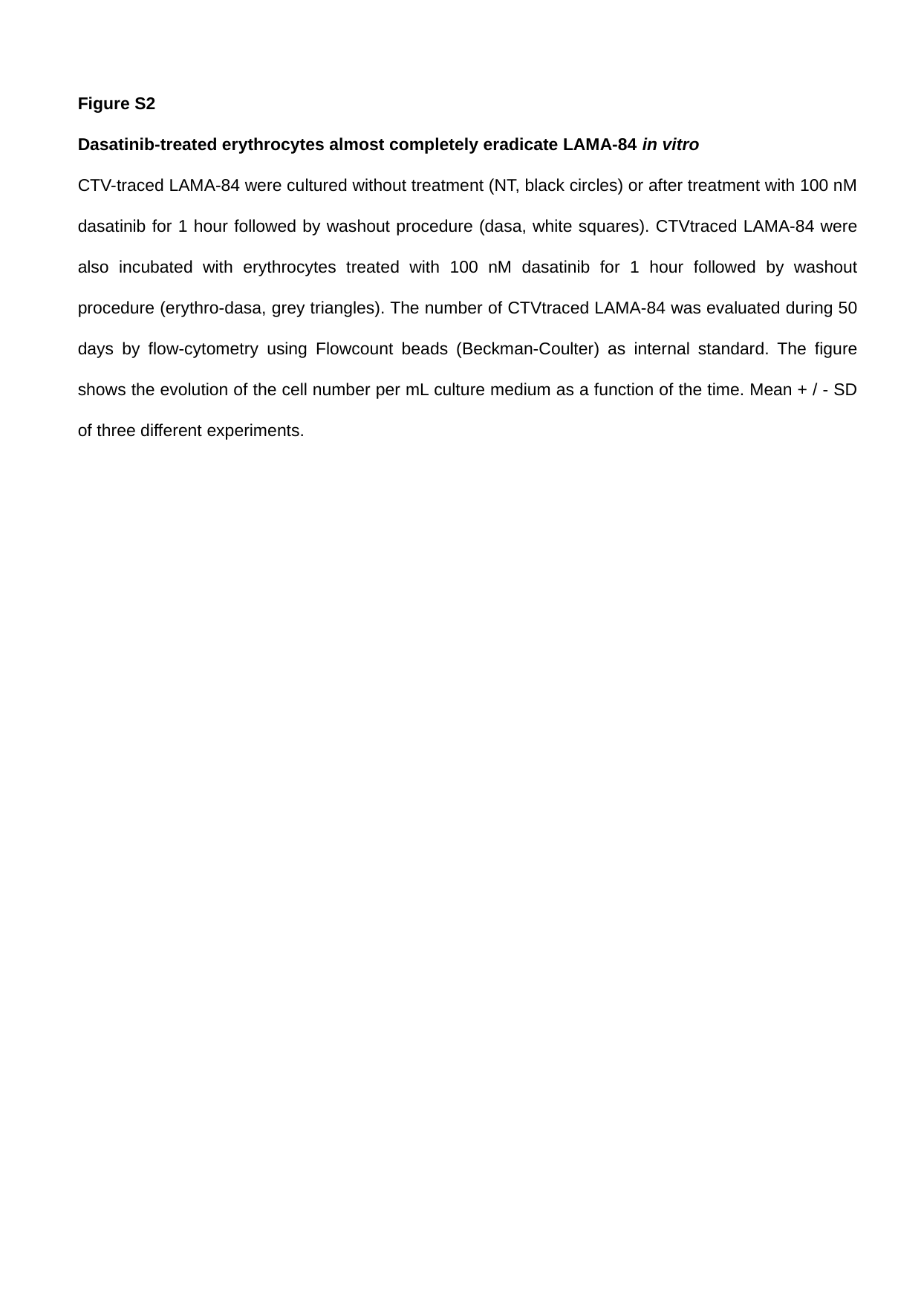

Figure S2
Dasatinib-treated erythrocytes almost completely eradicate LAMA-84 in vitro
CTV-traced LAMA-84 were cultured without treatment (NT, black circles) or after treatment with 100 nM dasatinib for 1 hour followed by washout procedure (dasa, white squares). CTVtraced LAMA-84 were also incubated with erythrocytes treated with 100 nM dasatinib for 1 hour followed by washout procedure (erythro-dasa, grey triangles). The number of CTVtraced LAMA-84 was evaluated during 50 days by flow-cytometry using Flowcount beads (Beckman-Coulter) as internal standard. The figure shows the evolution of the cell number per mL culture medium as a function of the time. Mean + / - SD of three different experiments.

## Slide 5
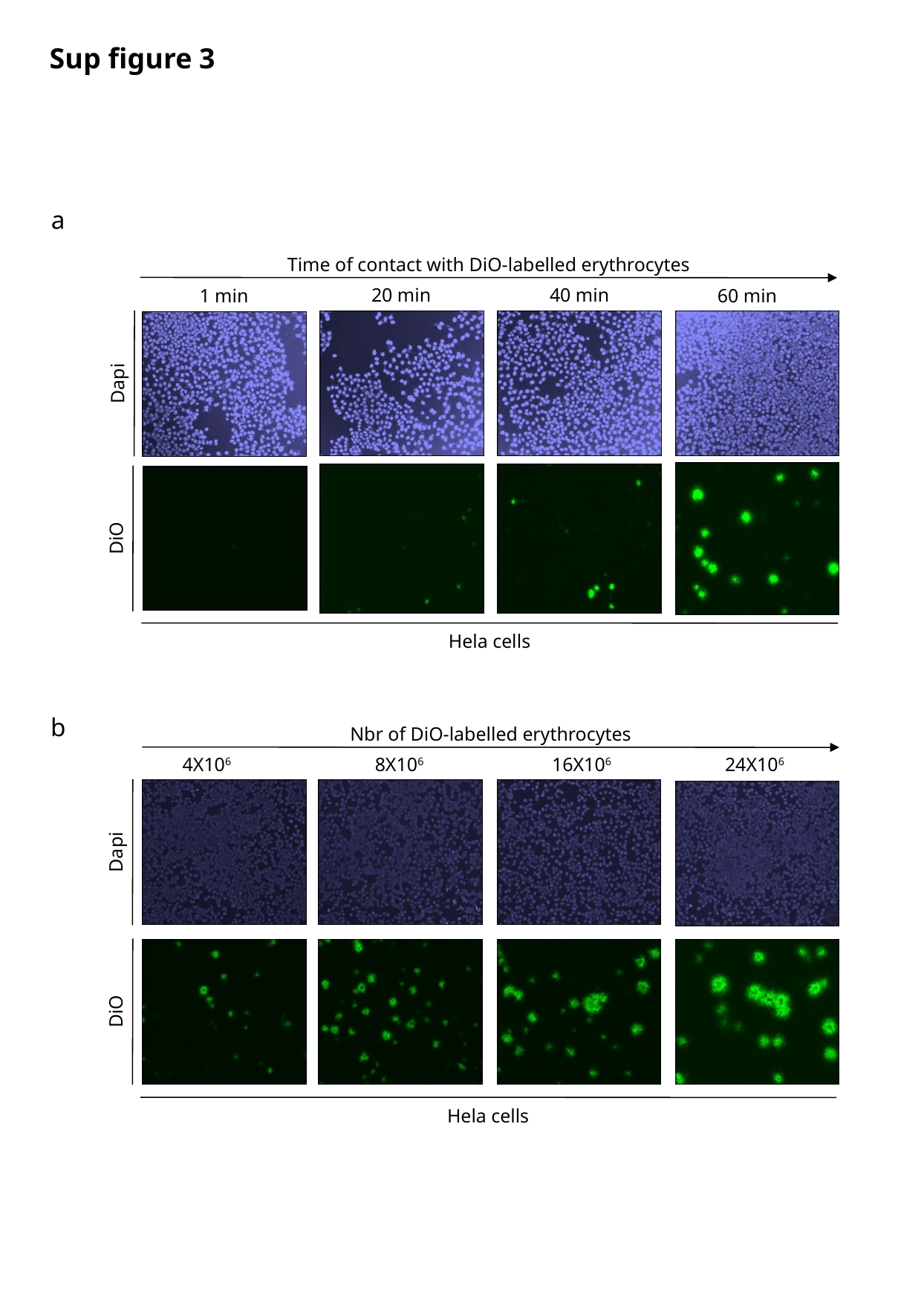

Sup figure 3
a
Time of contact with DiO-labelled erythrocytes
20 min
40 min
1 min
60 min
Dapi
DiO
Hela cells
b
Nbr of DiO-labelled erythrocytes
4X106
8X106
16X106
24X106
Dapi
DiO
Hela cells

## Slide 6
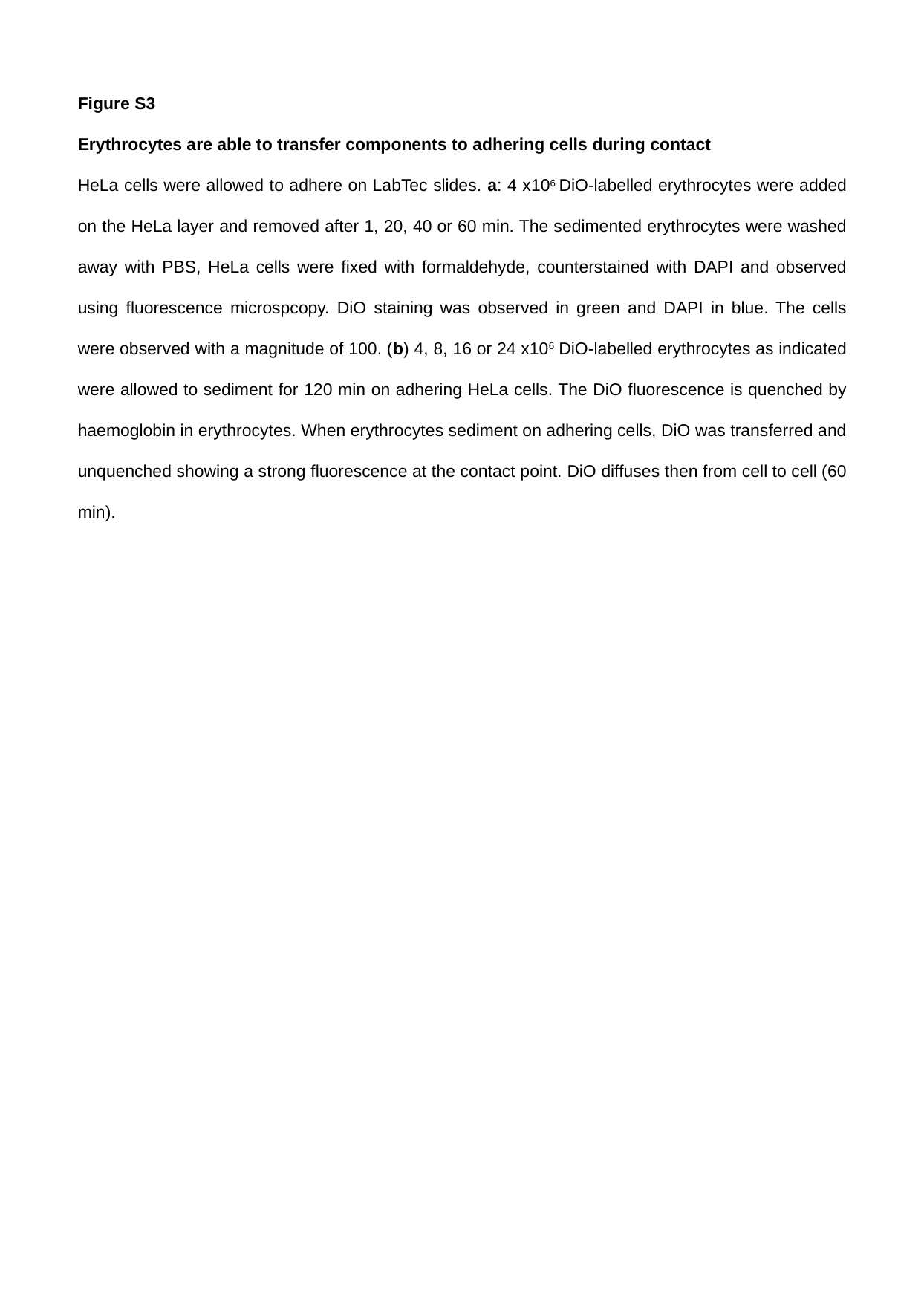

Figure S3
Erythrocytes are able to transfer components to adhering cells during contact
HeLa cells were allowed to adhere on LabTec slides. a: 4 x10^6^ DiO-labelled erythrocytes were added on the HeLa layer and removed after 1, 20, 40 or 60 min. The sedimented erythrocytes were washed away with PBS, HeLa cells were fixed with formaldehyde, counterstained with DAPI and observed using fluorescence microspcopy. DiO staining was observed in green and DAPI in blue. The cells were observed with a magnitude of 100. (b) 4, 8, 16 or 24 x106 DiO-labelled erythrocytes as indicated were allowed to sediment for 120 min on adhering HeLa cells. The DiO fluorescence is quenched by haemoglobin in erythrocytes. When erythrocytes sediment on adhering cells, DiO was transferred and unquenched showing a strong fluorescence at the contact point. DiO diffuses then from cell to cell (60 min).

## Slide 7
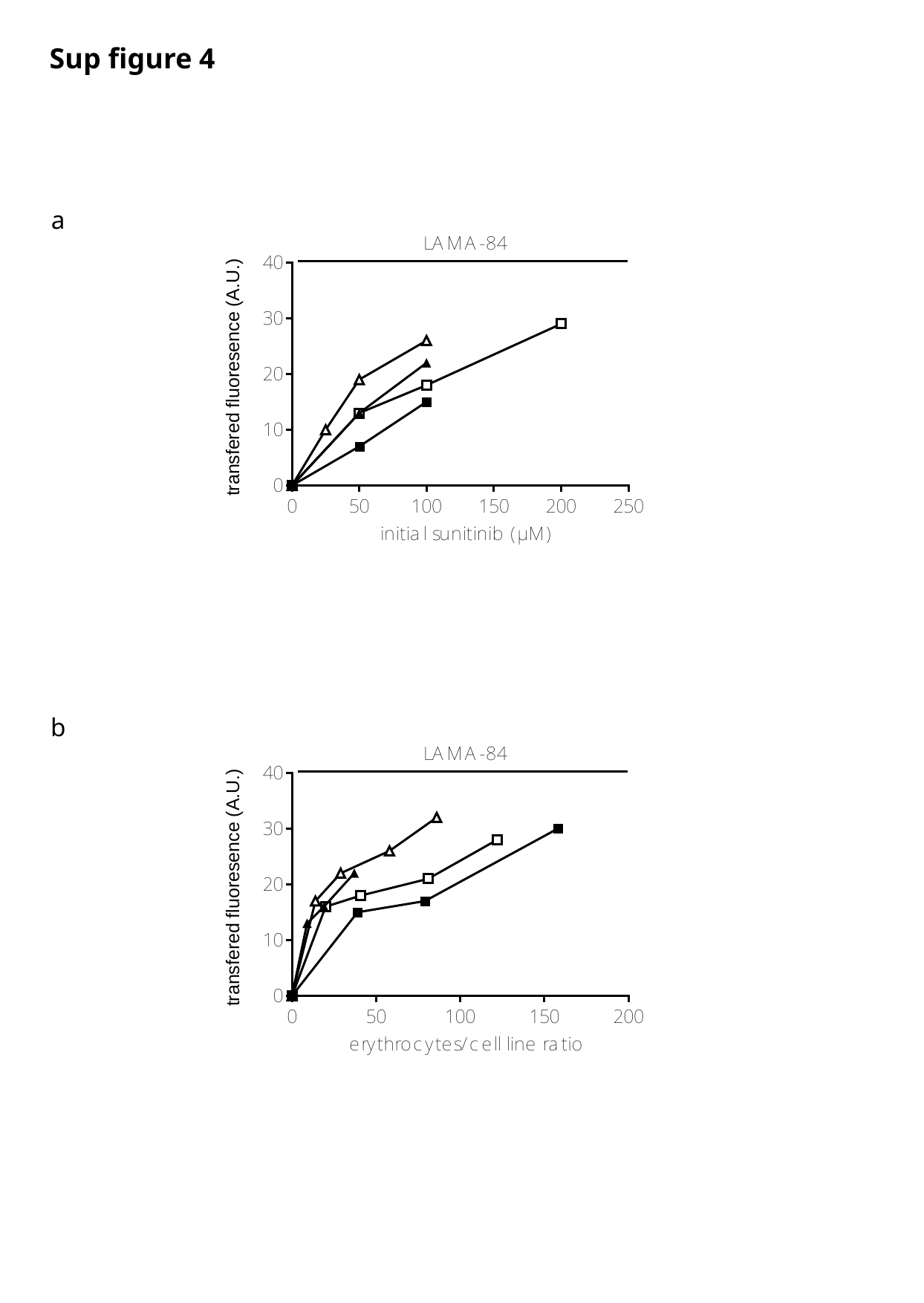

Sup figure 4
a
b

## Slide 8
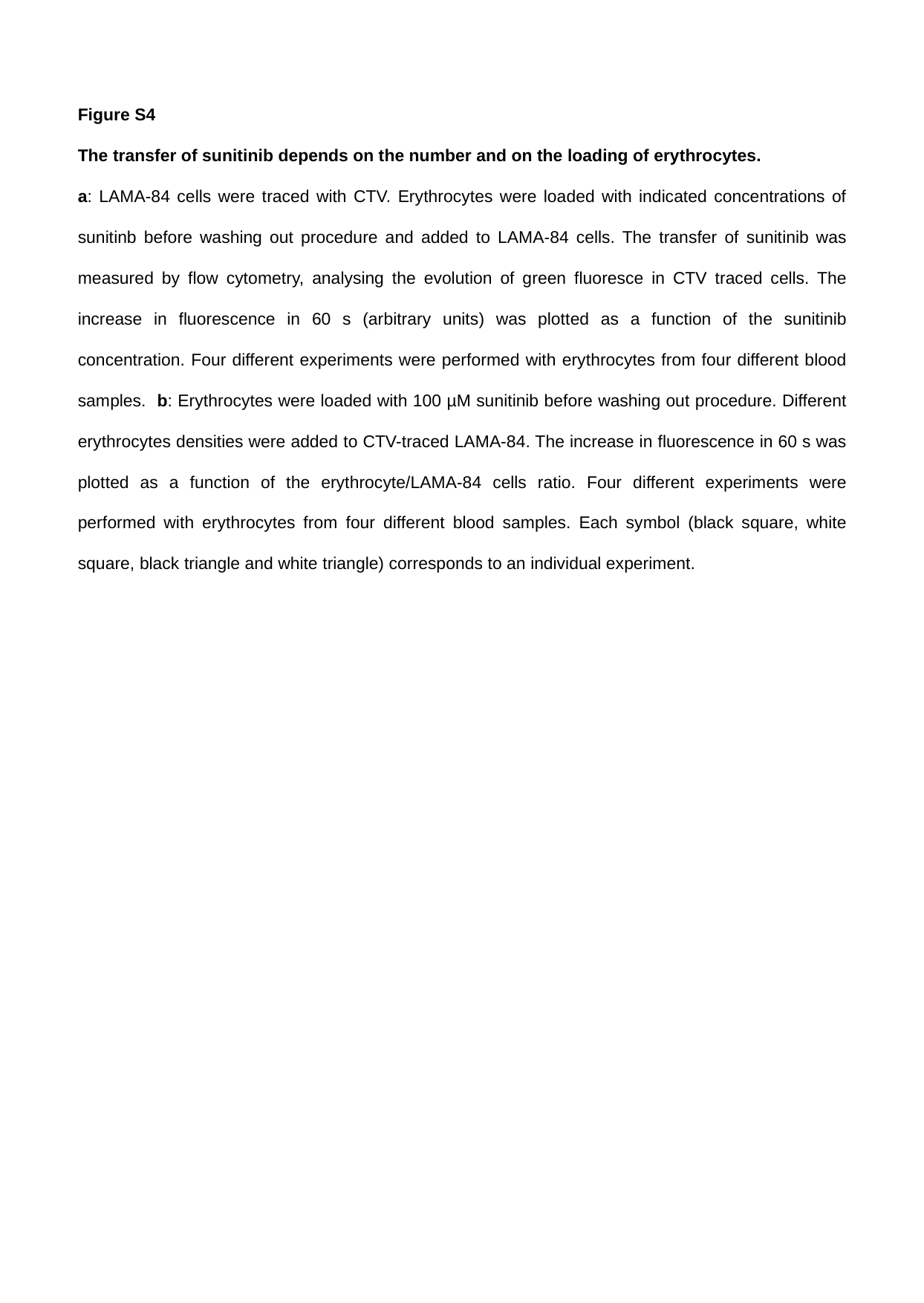

Figure S4
The transfer of sunitinib depends on the number and on the loading of erythrocytes.
a: LAMA-84 cells were traced with CTV. Erythrocytes were loaded with indicated concentrations of sunitinb before washing out procedure and added to LAMA-84 cells. The transfer of sunitinib was measured by flow cytometry, analysing the evolution of green fluoresce in CTV traced cells. The increase in fluorescence in 60 s (arbitrary units) was plotted as a function of the sunitinib concentration. Four different experiments were performed with erythrocytes from four different blood samples. b: Erythrocytes were loaded with 100 µM sunitinib before washing out procedure. Different erythrocytes densities were added to CTV-traced LAMA-84. The increase in fluorescence in 60 s was plotted as a function of the erythrocyte/LAMA-84 cells ratio. Four different experiments were performed with erythrocytes from four different blood samples. Each symbol (black square, white square, black triangle and white triangle) corresponds to an individual experiment.

## Slide 9
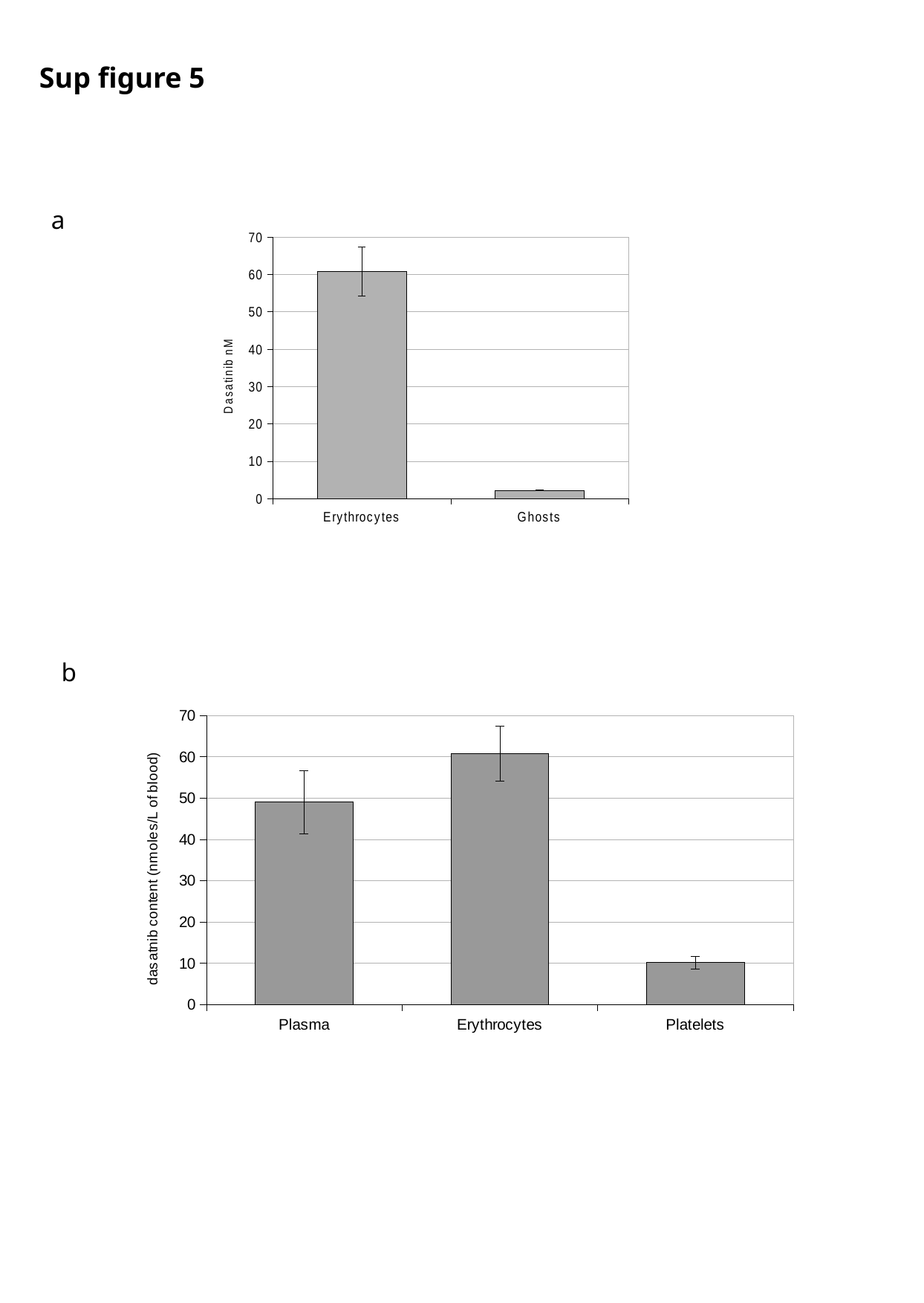

Sup figure 5
a
b

## Slide 10
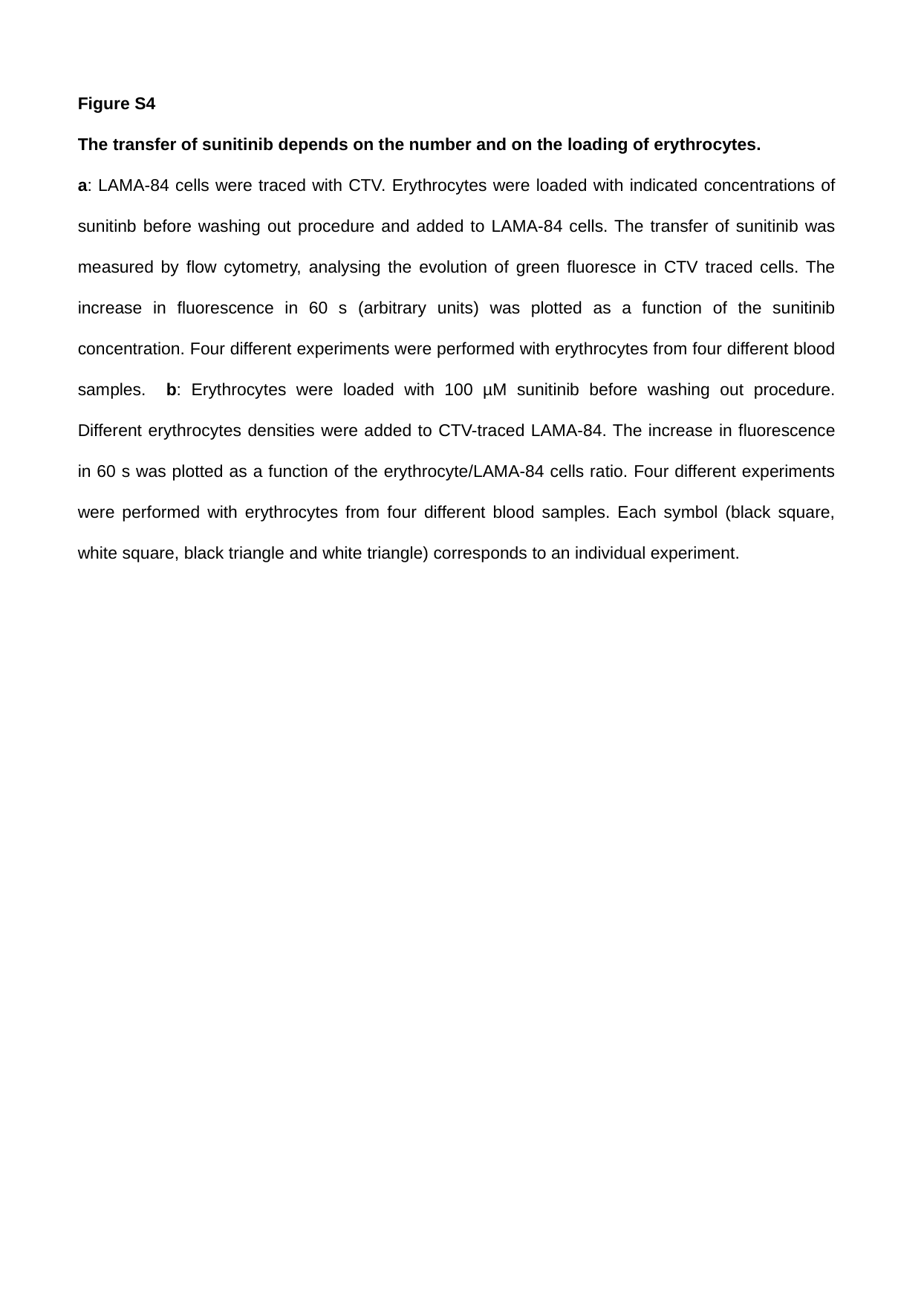

Figure S4
The transfer of sunitinib depends on the number and on the loading of erythrocytes.
a: LAMA-84 cells were traced with CTV. Erythrocytes were loaded with indicated concentrations of sunitinb before washing out procedure and added to LAMA-84 cells. The transfer of sunitinib was measured by flow cytometry, analysing the evolution of green fluoresce in CTV traced cells. The increase in fluorescence in 60 s (arbitrary units) was plotted as a function of the sunitinib concentration. Four different experiments were performed with erythrocytes from four different blood samples. b: Erythrocytes were loaded with 100 µM sunitinib before washing out procedure. Different erythrocytes densities were added to CTV-traced LAMA-84. The increase in fluorescence in 60 s was plotted as a function of the erythrocyte/LAMA-84 cells ratio. Four different experiments were performed with erythrocytes from four different blood samples. Each symbol (black square, white square, black triangle and white triangle) corresponds to an individual experiment.
